# Serum Concentration of Plant Sterol Oxidation Products (POP) Compared to Cholesterol Oxidation Products (COP) after Intake of Oxidized Plant Sterols: A Randomised, Placebo-Controlled, Double-Blind Dose–Response Pilot Study

**DOI:** 10.3390/nu11102319

**Published:** 2019-09-30

**Authors:** Yuguang Lin, Wieneke P. Koppenol, Diny Knol, Mario A. Vermeer, Harry Hiemstra, Silvia Friedrichs, Dieter Lütjohann, Elke A. Trautwein

**Affiliations:** 1 Unilever Research & Development Vlaardingen, Olivier van Noortlaan 120, 3133 AT Vlaardingen, The Netherlands (current and former†); yuguang.lin@hotmail.com (Y.L.); wienekekoppenol@gmail.com (W.P.K.); diny.knol@unilever.com (D.K.); mario@ver-meer.nl (M.A.V.); harry.hiemstra999@gmail.com (H.H.); 2 Institute of Clinical Chemistry and Clinical Pharmacology, University Clinics Bonn, Sigmund-Freud-Str. 25, D-53127 Bonn, Germany; silvia.friedrichs@ukbonn.de (S.F.); Dieter.Luetjohann@ukbonn.de (D.L.)

**Keywords:** autoxidation, cholesterol oxidation products (COP), nutrition study, oxysterols, phytosterols, plant sterols, plant sterol oxidation products (POP)

## Abstract

Plant sterols (PS) are oxidized to PS oxidation products (POP). This study quantified the change in serum POP compared to cholesterol oxidation products (COP) after the intake of increasing POP doses. This was a double-blind, randomized, placebo-controlled, dose‒response pilot study with healthy individuals in four groups (15 per group). The control group received products with no added PS or POP and treatment groups received daily 20–25 g margarine with added PS (mean 3 g/d) and two cookies (~28 g) for six weeks. Cookies delivered 8.7 (low-dose), 15.2 (medium-dose), or 37.2 (high-dose) mg/d POP. Fasting serum POP and COP were measured at the baseline, days 14, 28, and 42 in all participants and days 7, 21, and 35 in a subset. Sixty individuals completed the study; 52 were included in per protocol analysis. Serum POP increased with increasing POP intake and plateaued at dose >15 mg/d. Stabilized POP concentrations were (mean ± SD) 38.9 ± 6.9, 91.0 ± 27.9, 144.4 ± 37.9 and 203.0 ± 63.7 nmol/L, for control, low-, medium-, and high-dose POP groups, respectively. For all groups, the serum COP ranged from 213 to 262 nmol/L and the average POP/COP ratio was <1. Serum POP concentrations increased non-linearly, reaching stabilized concentrations in <7 days, and remained below COP concentrations after the intake of increasing POP doses.

## 1. Introduction 

Plant sterols (PS, also known as phytosterols) are natural compounds of plant origin that are similar in structure to cholesterol. They are effective at lowering low-density lipoprotein (LDL) cholesterol (LDL-C) concentrations by 7‒12.5% at daily PS intakes of 1.5‒3 g, as demonstrated in numerous clinical studies and summarized in several meta-analyses [1,2,3]. These efficacious levels of PS intake cannot be reached with conventional plant-based foods and therefore a variety of foods, such as spreads, margarines, and dairy-type foods like milk and yoghurt, with added PS, mostly in the form of PS esters, are commercially available [4].

Similar to cholesterol, the steroid ring of PS is susceptible to autoxidation, leading to the formation of PS oxidation products (POP) such as 7α/β-hydroxy (OH)-, 7-keto-, 5α,6α-/5β,6β-epoxy- and 3β,5α,6β-triol-derivatives of PS. POP are structurally similar to cholesterol oxidation products (COP). Both COP and POP are considered to be atherogenic [5,6,7]. In vitro and animal studies have reported the atherogenic potential of POP to be either similar to or less than COP [6,8,9,10,11]. Clear evidence of an atherogenic effect of POP in humans is lacking, but concerns around human exposure to POP have been voiced [6].

It has previously been debated whether the intake of foods with added PS may raise the atherosclerosis and cardiovascular disease (CVD) risk, mediated by the observed modest increase in plasma/serum PS concentrations after the intake of foods with added PS [12]. Observational studies that addressed such an association between plasma/serum PS and CVD risk have generated mixed evidence. The meta-analysis addressing this relationship, including 17 observational studies, found no significant association between circulating PS and CVD risk [13]. A putative mechanism for PS to be atherogenic relates to their oxidation susceptibility and the resulting formation of POP, which are suggested to be linked to atherogenicity, similar to COP [7]. 

POP are naturally present in PS-containing foods and are also generated during food manufacturing. Preparing foods through cooking and baking, especially using margarines with added PS, can further generate POP and hence increase their content in prepared foods [14,15]. Data on daily POP intake with a habitual diet are still scarce. In two studies, POP intake from non-heated margarines with added PS was 0.5 and 0.7 mg/d [16,17]. Estimated daily POP intakes, as calculated by Scholz et al. [15], ranged between 1.2 and 2.9 mg/d for non-heated foods with naturally occurring and added PS and from 24.5 to 29.6 mg/d for cooked foods prepared with PS-added margarine. Similarly, Lin et al. [14] estimated an upper POP intake of 48 mg/d based on a daily PS intake of 3 g/d (advised upper intake level) and a reported oxidation rate of PS (90% quantile) of 1.6%. Based on the measured POP contents of a variety of prepared foods such as meats, fish, vegetables, eggs, and potatoes, Lin et al. [18,19] estimated a daily POP intake of 7 mg/d (median; range 0.3‒64 mg/d), assuming one portion of such prepared foods is consumed per day. Taking these data together [14,15,18,19], the upper (90‒95% quantile) POP intake ranges between 25 and 48 mg/d, with the highest intake of 64 mg/d when using PS-added margarines for frying.

Plasma/serum POP and COP consist of 7α/β-OH-, 7-keto-, 5,6α/β-epoxy-, and triol-derivatives of PS and cholesterol (CH), respectively. Since plasma/serum 5,6α/β-epoxy- and triol-derivatives cannot be reliably measured, due to the autoxidation susceptibility of PS and CH during processing, recent studies focused on measuring only 7α/β-OH-PS/CH and 7-keto-PS/CH (together called 7-oxygenated-sterols). Therefore, and for better comparison between studies, plasma/serum POP are defined as the sum of 7α/β-OH-PS and 7-keto-PS and plasma/serum COP as the sum of 7α/β-OH-CH and 7-keto-CH.

A higher dietary POP intake will affect their circulating concentrations in blood. Plasma/serum concentrations of POP in individuals on habitual diets range between 10 and 33 nmol/L, with a mean value of about 19 nmol/L (median 12 nmol/L) [16,17,20,21,22]. For comparison, reported plasma/serum COP concentrations range from 184 to 266 nmol/L, with a mean of 227 nmol/L (median 231 nmol/L) [23,24], and hence are about 10 times higher than those of POP. 

Two human studies reported the dietary intake and the plasma/serum concentrations of POP following consumption of PS-enriched margarine [16,17]. Husche et al. [17] studied serum POP concentrations in 16 healthy individuals at the baseline and after four weeks of intake of 3 g/d PS and 505 µg/d POP from a PS-enriched margarine. The average total POP concentration (sum of all measured POP) was slightly (but not statistically significantly) increased from 12 nmol/L at the baseline to 16 nmol/L after intake of the PS margarine. This increase mainly resulted from an increase in 7β-OH-sitosterol of ~3 nmol/L. In another study, 43 healthy individuals consumed for four weeks 20 g/d of PS-enriched margarine, providing 3 g/d PS and 675 µg/d POP, or a control margarine without added PS and 98 µg/d POP [16]. Fasting POP concentrations did not differ following consumption of PS-added vs. control margarine (12 and 11 nmol/L, respectively) [16]. In a related publication, Baumgartner et al. [25] further reported that plasma POP concentrations remained relatively stable over time and during the different dietary interventions, and suggested the identification of ‘low vs. high oxidizers’ in their study population. In another study, postprandial plasma POP concentrations were investigated in 10 healthy individuals after a meal challenge containing 100 and 680 µg POP, respectively. Postprandial 7-keto-sitosterol and 7-keto-campesterol, which were the major POP measured, were not different from the control, while 7β-OH-sitosterol and 7β-OH-campesterol were slightly increased [26]. Notably, in all these studies, POP intakes were lower than 1 mg/d. 

Currently, there are no human data on plasma/serum POP concentrations after the consumption of foods that are prepared by cooking, such as shallow-frying foods using a PS-added margarine. Nor is it known how concentrations of POP would compare with those of COP under these conditions. 

Therefore, the aim of this study was to measure the increase in serum POP compared to COP concentrations after the dietary intake of POP over a range of daily consumption levels. The primary objective of the study was to quantify the change from baseline in serum total POP concentrations (sum of all individual POP measured) after a six-week intake of margarine with added PS plus increasing POP doses from cookies compared to a control group receiving margarine and cookies with no added PS or POP. A secondary objective was to quantify the corresponding changes from baseline in serum total COP concentrations (sum of all individual COP measured). Furthermore, serum POP and COP concentrations were compared by estimating the ratio of total POP/total COP. Lastly, as explorative outcomes the effects on serum PS concentrations and serum lipids, i.e., LDL-C, total cholesterol (TC), HDL-cholesterol (HDL-C) and triglycerides (TG) after a six-week intake of cookies with increasing POP contents and margarine with added PS compared to a control margarine with no added PS or POP were assessed.

## 2. Materials and Methods 

The study took place from August to December 2017 at the Charité Research Organisation GmbH, Berlin, Germany. The study was conducted in accordance with applicable laws and regulations, including the International Conference on Harmonization (ICH), Guideline for Good Clinical Practice (GCP) and the ethical principles that have their origins in the Declaration of Helsinki. The protocol, informed consent, advertisements, and protocol amendments were approved by the ethics committee at Charité Hospital Berlin, Germany. Written informed consent was obtained from all study participants. The study was registered at clinicaltrials.gov as NCT03312816.

### 2.1. Study Population

The study population consisted of healthy men and women. Study participants were recruited by advertisements in local newspapers and on the Internet. Individuals were eligible to be enrolled in the study when they met the following main predefined inclusion criteria: aged 40–75 years, having a body mass index (BMI) >18 and <35 kg/m^2^, TC concentration at screening ≥ 5.0 and ≤ 8.0 mmol/L, blood pressure, heart rate, haematological and clinical chemical parameters within the normal reference range, willing to comply with the study products intake and the dietary restrictions of the study, agreeing to be informed about medically relevant personal test results by a physician, and having signed the informed consent. Participants were excluded if they had been recently (<6 months) diagnosed with cardiovascular event(s) or systemic inflammatory conditions, used PS/stanol-enriched foods or supplements in the three months prior to screening and/or during the study, used over-the-counter and/or prescribed medication that may interfere with study measurements (i.e., statins, ezetimibe, fibrates, diabetes drugs, angiotensin receptor blockers (ARB), and ACE inhibitors), were pregnant or lactating women, reported alcohol consumption >14 units/week (female) or >21 units/week (male), reported intense sporting activities of >10 h/week, had weight loss or gain of 3 kg or more during a period of six months prior to screening, reported participation in another nutritional or biomedical study three months prior to screening and/or during the study, were currently smoking or had been a non-smoker for less than six months and reported use of any nicotine-containing products in the six months prior to screening and/or during the study, and reported intolerance to study products provided during the study. Being an employee of Unilever or the Charité Research Organisation was also an exclusion criterion. 

### 2.2. Study Design

The pilot study used a randomized, double-blind, placebo-controlled, parallel dose‒response design with four study groups. Participants were randomly allocated using a computer-generated random allocation to treatment arms with the number of individuals equally divided across the groups to consume 0 (control), 12.5 (low dose), 25 (medium dose), or 50 mg/d (high dose) of added POP for six weeks. These intakes reflect the actual range of POP intakes from foods prepared by cooking with PS-added margarines [18,19]. Eligible participants were stratified according to gender, with the ratio of women/men as equal as possible for all groups.

Blood samples were taken at the baseline (days −2 and 0), at intermediate time points (days 14 and 28) and at the end of the intervention (day 42) from all study participants. Furthermore, the study population was divided into three subsets (each subset *n* = 20; five subjects from each intervention group). From these subsets of participants, an additional blood sample was taken either on day 7, 21, or 35. Serum POP and COP were measured in samples taken on days −2, 0, 14, 28, and 42 and in subsets on days 7, 21, and 35; serum PS were analysed in samples taken at days 0 (baseline) and 42 and serum lipids were analysed in samples taken at days −2, 0 (data pooled as baseline) and 28 and 42 (data pooled as end of intervention). On all test days, participants came to the study centre in a fasted state (10 h of neither food nor drink except water) and received breakfast after each fasting blood sampling. Body weight, health and wellbeing, compliance with study products intake, use of concomitant medication, and adverse events (AEs) were monitored throughout the study.

### 2.3. Study Intervention and Study Products

Study participants were instructed and regularly reminded to minimise changes to their habitual diet and lifestyle, including their usual physical activity habits, during the entire study period. They were further instructed to refrain from consuming other foods or supplements with added PS/stanol or other foods and supplements claiming to lower cholesterol throughout the study period. Participants were given a personal diary that clearly described all restrictions and in which compliance, i.e., daily intake of study products and concomitant medication use, had to be self-recorded. All unopened study products had to be returned to the study centre at the end of the intervention on day 42 and returned study products were counted. Compliance was predefined as a minimum study product intake of 90%.

During the intervention period, study participants were provided with two study products: a margarine with 35% fat without and with 9.4% added PS (in the form of PS esters) and cookies without or with increasing doses of POP. Two cookies and two portions of margarine were consumed per day. The PS and POP contents in the margarine and cookies are presented in Table 1.

Margarine: Margarines were provided as either 10 g or 12.5 g portion packs, produced in one batch at Unilever Baking, Cooking and Spreads (BCS) Research & Development (R&D) Vlaardingen, the Netherlands. Participants were asked to refrigerate the margarines at home. Cooking, baking, or frying with the margarine was not allowed. Each day, participants consumed two portions (i.e., 20 to 25 g/d) margarine over two main meals, i.e., breakfast, lunch, and/or dinner. Participants in the low-dose POP group received 25 g PS-added margarine (2.3 g added PS) and those in the medium-dose POP group received 20 g PS-added margarine (1.8 g added PS). The control group and the high-dose POP group received 25 g of the regular margarine without added PS.

Cookies: Cookies were baked in three batches at the industrial bakery of Sonneveld Group, Papendrecht, the Netherlands, according to a defined recipe based on 100 g fat (either the POP-rich fat (see below) alone or combined with a commercial baking margarine), 150 g flour, and 50 g sugar per 20 cookies. Study participants consumed daily two cookies of ca. 15 g each as part of a main meal, e.g., with either lunch or dinner. The control cookies contained trivial amounts of PS and POP.

### 2.4. Production of a POP-Rich Fat

A POP-rich fat used to deliver the increasing doses of POP in cookies was produced through shallow-frying potatoes with a margarine with 22.5% added PS (in the form of 37.5% PS esters) in a canteen setting. After shallow-frying, the potatoes were discarded and the residual fat (RF) left in the frying pan was collected. Ca. 36 kg of PS-added margarine and ca. 150 kg potatoes were used to generated 13.7 kg POP-rich fat. The PS and POP contents of the POP-rich fat used for baking cookies were 31 g and 555 mg per 100 g fat, respectively. POP doses studied were ultimately defined based on volume and POP content of the generated POP-rich fat to produce the required number of cookies for the six-week study duration.

### 2.5. Analytical Procedures/Methods

Throughout the study, blood was withdrawn into a serum gel Monovette^®^ and kept at room temperature for at least 30 min before centrifuging at 1300 x g for 15 min at room temperature. To avoid autoxidation, 15 μL of 3,5-di-tert-butyl-4-hydroxytoluene (BHT; 5 mg/mL methanol, w/v) were added to 1.5 mL serum. The resulting serum was stored at −20 °C until analysed at the completion of the study. All samples from each subject were analysed within the same analytic run.

POP and COP analysis: POP including 7α-hydroxy (OH)-campesterol, 7α-OH-sitosterol, 7β-OH-campesterol, 7β-OH-sitosterol, 7-keto-campesterol and 7-keto-sitosterol and COP including 7α-OH-, 7β-OH- and 7-keto-CH were extracted after alkaline hydrolysis with dichloromethane. COP and POP were further separated by solid-phase extraction. POP and COP were derivatized into their corresponding trimethylsilyl-ethers and quantified by gas-chromatography‒mass selective detection (GC-MS) using corresponding deuterium labelled COP or POP as internal standards (isotope dilution methodology), based on the method described by Husche et al. [17]. 

PS analysis: The PS campesterol and sitosterol, as well as TC (used to calculate cholesterol-standardised concentrations from the same sample), were measured by GC-FID using 5α-cholestane as the internal standard [27]. 

All POP, COP, and PS analyses were carried out at the Laboratory for Special Lipid Diagnostics/Centre Internal Medicine at the Institute for Clinical Chemistry and Clinical Pharmacology of the University of Bonn, Germany.

Serum lipids: TC and TG were measured using enzymatic reagents from Diasys (Holzheim, Germany) and were calibrated using secondary standards from Roche Diagnostics (Mannheim, Germany). LDL-C and HDL-C were measured using homogeneous enzymatic assays and secondary standards from Diasys (Holzheim, Germany). All measurements were performed on an Olympus AU680 automated analyser (Beckman Coulter, Brea, CA, USA) and carried out at the Clinical Institute of Medical and Chemical Laboratory Diagnostics, Medical University of Graz, Austria.

### 2.6. Sample Size Calculation, Predefined Outcomes, and Statistical Analysis

#### 2.6.1. Sample Size 

The study was an exploratory study aimed to generate data on serum POP concentrations over time after daily intake of increasing dietary POP doses. It was not possible to perform a formal power calculation, as prior estimates of the parameters needed for modelling changes in serum POP concentrations over time was lacking. Typically, for exploratory studies, a minimal number of 12 study participants per study arm is recommended as a ‘rule of thumb’ [28]. Therefore, for the study with four arms, i.e., a control and three intervention (increasing doses of POP) groups, a total of 48 subjects was needed. To account for possible dropouts and non-compliance, 60 individuals (15 per study arm) were enrolled into the study.

#### 2.6.2. Outcomes

The pre-specified primary outcome measures were the sum (Σ) of individual serum POP concentrations, secondary outcomes were Σ of serum COP concentrations and exploratory outcomes were serum PS concentrations and serum lipids, i.e., LDL-C, TC, HDL-C, and TG.

#### 2.6.3. Determination of Steady State Concentrations for POP and Changes from Baseline 

For the estimation of the change from the baseline relative to the control, the individual stabilised concentrations of Σ of POP concentrations after the intake of increasing doses of dietary POP were defined as estimated steady-state concentrations (Css). Stabilised ΣPOP concentration refers to the steady-state concentration as modelled over the six-week intervention. ΣPOP was calculated by including 7α-OH-, 7β-OH- and 7-keto- derivatives of sitosterol and campesterol, respectively. When the measured individual POP were lower than the limit of quantification (LOQ) and limit of detection (LOD) as reported by Husche et al. [17], these were taken into account for the estimation of the corresponding individual POP.

The change of serum ΣPOP over time can be described by a mono-exponential increase based on a well-known pharmacological model [29,30]. This model also includes a baseline for ΣPOP and is defined as follows:

ΣPOP (time) = baseline + Css (1 − exp [log (1 − f)/Tf × time]),(1)

where ΣPOP(time) describes the evolution of the observed ΣPOP over time, baseline the pre-intervention concentration ΣPOP (estimated), Css is the asymptotic steady state concentration of ΣPOP for each intervention group (estimated) and Tf is the “time to reach fraction*100% of Css”; f was set to 0.9, i.e., T0.9 represents the time to reach 90% of estimated Css. 

This mono-exponential curve of ΣPOP with baseline was fitted with PROC NLMIXED (v9.4, SAS Institute Inc., Cary, NC, USA) using a non-linear mixed modelling approach. This approach takes into account the between-subject variability via introducing random effects for baseline and Css. The time to reach 90% of the Css (T0.9) entered the model as a fixed effect only, as a random effect resulted in very instable models. Furthermore, for the control group it was assumed that the ΣPOP levels did not change over time and remained at the baseline level given the very low POP intake. This model yields specific estimates for baseline and Css for each study participant. The adequacy of the model was further explored via the analysis of standardized residuals. Of note, the steady state concentration of ΣPOP, i.e., including the baseline, is the sum of the subject-specific Css and the subject-specific baseline, hence the change from baseline steady state concentration of ΣPOP is equal to the subject-specific Css. 

For the three intervention groups receiving increasing dietary POP intakes (i.e., excluding the control group), the estimated change from baseline Css was also used to assess dose proportionality, i.e., whether doubling the daily POP dose would result in a doubling of Css, which was determined using a power ANOVA (analysis of variance) model [31].

**Figure 1:**
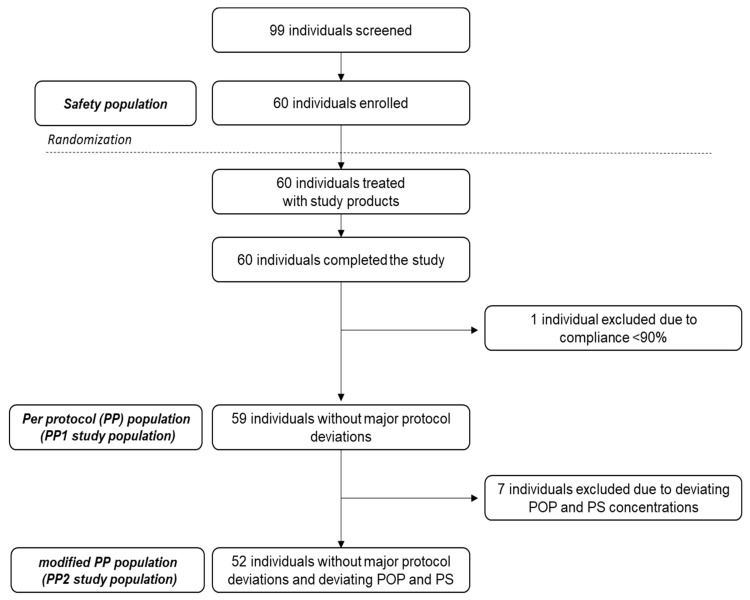
Flow diagram depicting the flow of participants throughout the study. POP: plant sterol oxidation products, PS: plant sterols.

Due to observed deviations in the POP concentrations of seven study participants (see Figure 1), a mono-exponential curve could not be reliably used to determine the steady state concentration. Therefore, the change in ΣPOP over time was also described with a “piecewise” linear mixed regression model. This “piecewise” linear mixed regression model assumes for the POP intake groups that the baseline concentration of ΣPOP up to day 0 is constant and during increased exposure from the intervention increases linearly up to a breakpoint, after which ΣPOP concentrations remain constant. For the control group, it was assumed that the baseline ΣPOP concentration remains constant over time. 

The between-subject variability was considered by introducing random effects for baseline and the post-breakpoint concentration. The adequacy of the model was further explored via the analysis of standardised residuals. The post-breakpoint concentration of ΣPOP, deemed to be the equivalent of the Css of ΣPOP, was derived with the mono-exponential model. 

#### 2.6.4. Determination of Css for COP and Change from Baseline

Determination of the steady state of serum ΣCOP for each of the four study groups assumed that the ΣCOP concentrations did not change over time and remained at their baseline level. This was modelled using a simple model that only took between-subject variability into account by introducing random effects for the baseline. This model yielded specific estimates for Css serum ΣCOP for each study participant.

#### 2.6.5. Calculating the Ratio of Serum ΣPOP/ΣCOP

The statistical analysis made use of the two observations for each study participant, i.e., the subject-specific Css of ΣPOP (including baseline) and ΣCOP. These observations were treated as response variables in an ANOVA model with the predictors ‘intervention group’ (categorical variable with four levels: control, low, medium, high POP intake), ‘compound’ (categorical variable with two levels: Css POP, Css COP), and the interaction between compound and intervention group and possibly subject-specific characteristics such as gender (categorical variable with two levels: male, female), age, and body weight as possible covariates. The relation between these observations on the same study participant was incorporated in the model. 

For log transformed variables, the LSmeans for differences extracted from the interaction were back transformed to the mean ratio estimate and its associated 95% confidence interval (CI) for each of the intervention groups.

#### 2.6.6. Change from the Baseline, Compared to the Control, of Serum PS and Serum Lipids

Serum lipid concentrations (TC, LDL-C, HDL-C and TG) at the baseline (the average of samples two days apart) and two samples at the end of intervention (days 28 and 42) after intervention were averaged. All averaged blood lipid concentrations (baseline as well as post-intervention) were subsequently log-transformed. The effects of intervention on serum lipids were analysed with an ANCOVA model using post-intervention lipids on a log-scale as response. The model always included baseline (on log scale) and intervention group. Gender, age, body weight, and the interaction between intervention group and gender were included in the model as covariates, but were dropped from the model if they did not contribute to the model based on the Bayesian Information Criterion as a goodness of fit criterion. The effect of the interventions was reported as an estimate of the relative change in serum lipids (expressed as a percentage and an associated 95% CI) using the control intervention as a reference. In addition, the results were also expressed as absolute changes plus associated 95% CI, based on a transformation method [32]. A similar analysis was performed with the low, medium, and high POP intake intervention groups combined into one intervention group. The serum PS concentrations measured at the baseline and at the end of the intervention (both single measurements) were analysed in a similar way as described for the serum lipids.

As the PS intake in the three POP dose groups was similar, the effects of PS intake on serum PS concentrations and serum lipids compared to the control were assessed by combining the three POP dose groups.

All analyses were performed with the statistical software package SAS version 9.4 (SAS Institute Inc.). For all statistical analyses a two-sided significance level of 0.05 was used.

## 3. Results

### 3.1. Study Participants and Dietary Compliance 

In total, 99 individuals were screened, of whom 60 were selected and randomised into the study. All 60 study participants completed the study with no dropouts. One individual was excluded for per protocol (PP) analysis due to low (<90% as predefined) compliance with cookie intake; hence 59 individuals were available for PP analysis of the primary study outcome (defined as PP1 study population). Further data reviewing (during data analysis) identified seven study participants, two in the medium-dose and five in the high-dose POP groups, with no or a lower than expected increase in serum POP. These individuals also had close to zero increases in serum PS (campesterol and sitosterol) compared to the baseline concentrations. Regardless of the cause (e.g., individuals were noncompliant with cookie intake and/or non- or low responders to PS and POP intakes), to avoid bias and to present the most conservative interpretation, these seven study participants were excluded; the remaining study population of 52 is therefore defined as the PP2 study population (Figure 1). 

The results obtained from the PP2 study population are considered as the main study outcome; those of the PP1 study population are presented for comparison.

An overview of the study participants’ characteristics at screening is provided in Table 2. Of the 60 study participants, 28 were men and 32 women. 

Compliance with study products intake, based on assessment of the personal diaries and returned study products, was overall high, except for one individual in the control group, who was excluded due to low (<90%) compliance.

### 3.2. Dietary Intakes of POP and PS

POP intake: The actual POP intakes were about 30–40% lower than planned due to the lower weight of the cookies (13–14 g/cookie instead of the designed 15 g) and because the measured POP contents in the fat extracted from the cookies was lower than that of the POP-rich fat used to bake the cookies. Therefore, the actual POP intakes were 8.7, 15.2, and 37.2 mg/d, with the majority being delivered through the consumption of two cookies per day. Margarine intake contributed only <0.5 mg to the daily POP intake.

PS intake: Both margarine and cookies contributed to the daily PS intake, with an average PS intake of 3 g/d (2.7 g/d in the high-dose POP group, 3.0 g/d in the low-dose POP group, and 3.2 g/d in the medium-dose POP group). While the main intake of PS in the low-dose and medium-dose POP groups came from the margarine (2.34 and 1.87 g, respectively), cookies contributed 2.69 g to the daily PS intake in the high-dose POP group.

### 3.3. Adverse Events

The study products were well tolerated by the study participants. Adverse events (AEs) were monitored at all visits. In total, 16 AEs were reported in 12 study participants; 10 AEs (in eight individuals) occurred in the groups received the increasing POP doses, and six (in four individuals) in the control group. All AEs were not or unlikely to be related to study product intake and/or procedures. The most common AEs were common cold and headache. No serious AEs occurred.

### 3.4. Individual Variation of Serum POP Concentrations in Response to Dietary POP Intake

**Figure 2:**
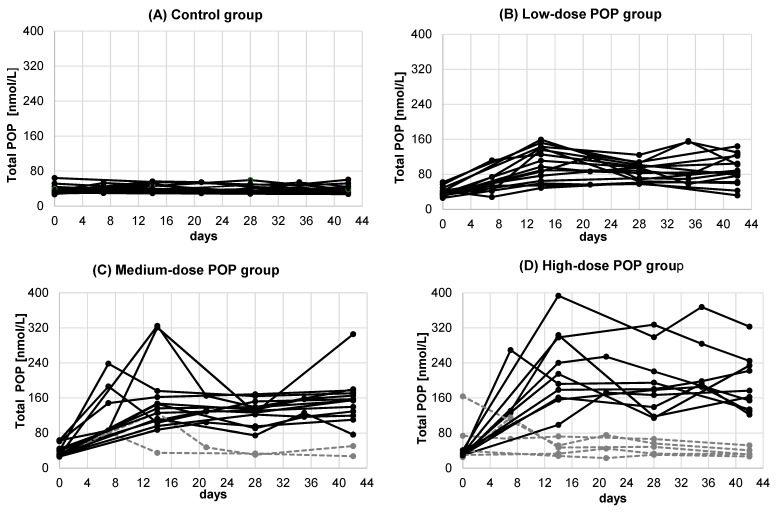
Individual serum POP concentrations over the six-week intervention period in study participants consuming increasing doses of POP (PP1 study population). (**A**) control group, (**B**) low-dose POP group, (**C**) medium-dose POP group, (**D**) high-dose POP group. The seven individuals in the medium dose (**C**) and high-dose (**D**) POP groups who were excluded from the PP1 study population (for details, see under 3.1) are marked in grey with dashed lines. Total POP refers to the sum of all individual POP measured including 7-keto and 7α- and 7β- OH-derivates of PS.

Individual changes in serum POP concentration in all 59 study participants (PP1 study population) who consumed increasing doses of dietary POP for six weeks are shown in Figure 2. 

This figure also highlights (dashed, grey) the seven study participants who showed no or lower than expected increases in serum POP and PS concentration compared to the rest of the corresponding POP dose group and were therefore excluded from the PP1 study population; hence the PP2 study population consists of 52 subjects (black curves). Of the 52 subjects, 27 were men (seven in the placebo group, seven in the low-dose group, seven in the medium-dose group, and five in the high-dose POP groups) and 25 women (six in the placebo group, eight in the low-dose group, six in the medium-dose group, and five in the high-dose POP group).

### 3.5. Time to Reach Steady State Serum POP Concentrations after POP Intake

**Figure 3:**
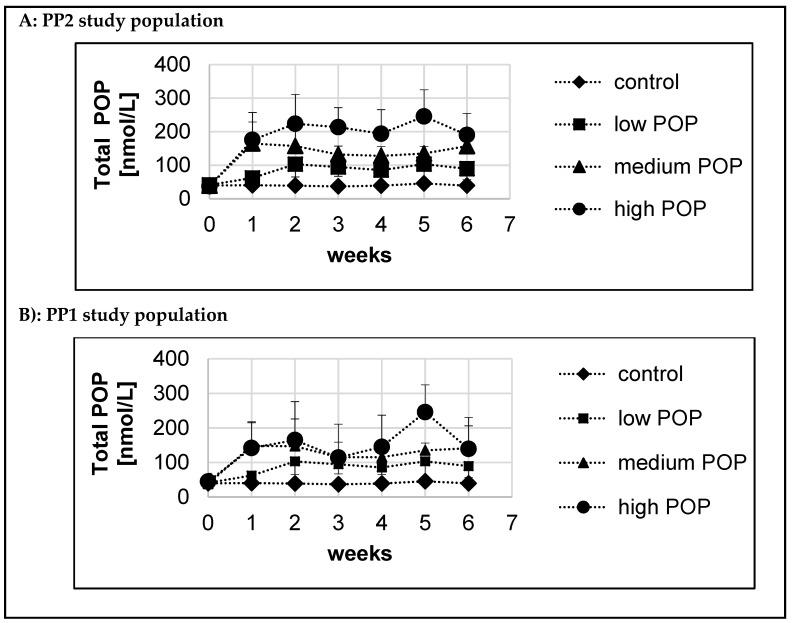
Serum total POP concentrations over time during the six-week dietary intake of increasing doses of POP. Data are presented as mean ± SD. Total POP refer to the sum of all individual POP measured including 7-keto and 7α- and 7β-OH derivates of PS. Panel A: PP2 study population(**A**). The number of study participants in the different groups and weeks are: Control: weeks 0, 2, 4 and 6 *n* = 14; weeks 1 and 5 *n* = 5; week 3 *n* = 4; Low POP dose: weeks 0, 2, 4 and 6 *n* = 15; weeks 1, 3 and 5 *n* = 5; Medium POP dose: weeks 0, 2, 4 and 6 *n* = 13; weeks 1 and 3 *n* = 4; week 5 *n* = 5; High POP dose: weeks 0, 2, 4 and 6 *n* = 10; week 1 *n* = 3, week 3 *n* = 2 and week 5 *n* = 5. Serum POP concentrations over time were described by a mono-exponential increase based on a pharmacological model as described in detail under statistical analyses. Based on modelling the time to reach a 90% steady state concentration (Css) was 5 d. Panel B: PP1 study population (**B**). The number of study participants in the different groups and weeks are: Control: weeks 0, 2, 4 and 6 *n* = 14; weeks 1 and 5 *n* = 5; week 3 *n* = 4; low POP dose: weeks 0, 2, 4 and 6 *n* = 15; weeks 1, 3 and 5 *n* = 5; Medium POP dose: weeks 0, 2, 4 and 6 *n* = 15; weeks 1, 3 and 5 *n* = 5; High POP dose: weeks 0, 2, 4 and 6 *n* = 15; weeks 1, 3 and 5 *n* = 5. Serum POP concentrations over time were described by a “piece wise” linear mixed regression model. This model assumes for the POP intake groups that baseline concentration of ΣPOP up to day 0 is constant and increases linearly thereafter up to a breakpoint, after which ΣPOP concentrations remains constant over time after this time point. For the control group it was assumed that the concentrations remained constant over time. Based on modelling (see statistical analysis) the position of the breakpoint for the POP intake groups as determined was 6.9 d.

Mean serum POP concentrations over the six-week intervention period in all groups for both the PP1 and PP2 study populations are presented in Figure 3A,B. In the control group, serum POP concentrations did not change over time and remained around the baseline concentrations. In the medium-dose and high-dose POP groups, serum POP concentrations in the PP1 study population were generally lower than those in the PP2 study population. 

For each study participant the estimated Css of serum POP after intake of increasing doses of dietary POP was calculated based on modelling, as described in detail in the Section 2.6.3. This model allowed us to estimate the time to reach 90% of Css of serum POP, providing a parameter for how fast increased dietary POP intake resulted in a stabilised serum POP concentration. For the PP2 and PP1 study populations, the estimated time to reach 90% of Css of serum POP was 5.0 d and 6.9 d, respectively.

### 3.6. Stabilised Serum POP and COP Concentrations and Change from Baseline after Dietary Intake of Increasing POP Doses

**Figure 4:**
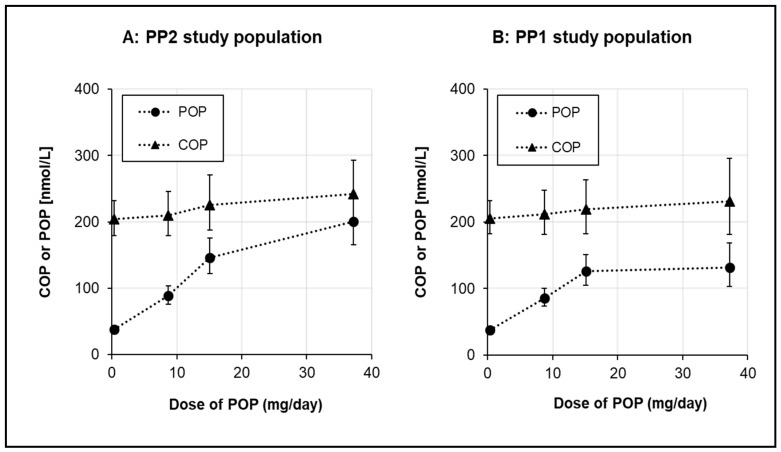
Serum total COP concentrations and stabilized concentrations of serum total POP with increasing dietary intakes of POP. Data are presented as mean plus 95% CI. Total COP and POP refer to the sum of all COP and POP measured including 7-keto and 7α- and 7β- OH derivates of PS and cholesterol, respectively. Stabilized total POP concentration refers to the steady-state serum concentration as modelled over the six-week intervention. For PP2 study population (**A**): *n* = 52 (*n* = 14 in control, *n* = 15 in low POP dose, *n* = 13 in medium POP dose and *n* = 10 in high POP dose group) For PP1 study population (**B**): *n* = 59 (*n* = 14 in control, *n* = 15 in low POP dose, *n* = 15 in medium POP dose and *n* = 15 in high POP dose group)

The stabilised (steady-state concentration modelled over the six-week intervention) serum total POP concentrations with increasing dietary POP intake are presented in Figure 4. For comparison, serum COP concentrations are also shown. In the PP2 study population, the stabilised serum POP concentrations (mean ± SD) were 38.9 ± 6.9, 91.0 ± 27.9, 144.4 ± 37.9 and 203.0 ± 63.7 nmol/L, for the control, low-dose, medium-dose and high-dose POP groups, respectively. The respective changes from the baseline were −0.45, 50.2, 105.9, and 166.2 nmol/L. Serum POP concentrations increased with increasing POP intake, but the analysis of dose proportionality revealed that the increase was non-linear and appeared to plateau at a dose > 15 mg/d.

Despite the fact that the serum COP concentration was somewhat higher in the high-dose POP group than in the control group, the change from baseline of COP in the high-dose POP group was −6.56 nmol/L (Table 3). Taken together, increasing dietary POP intakes resulted in an increase in the serum POP concentration, but had no effect on the serum COP concentration. 

Post hoc analysis of study participants, separated by sex, indicated that while the POP dose-dependent increases in serum POP concentrations were similar between men and women, serum COP concentrations were overall lower in women than in men.

Relative to the PP2 study population, the PP1 study population had lower stabilised serum POP concentrations in the medium-dose and high-dose POP groups (Figure 4). The changes in serum POP were ‒0.45, 50.2, 93.1, and 106.6 nmol/L in the control, low-dose, medium-dose, and high-dose POP groups, respectively. POP concentrations increased steadily up to the medium-dose (15.1 mg/d) POP intake, while the high-dose (37.2 mg/d) POP intake only modestly further increased serum POP concentrations in the PP1 study population.

**Figure 5:**
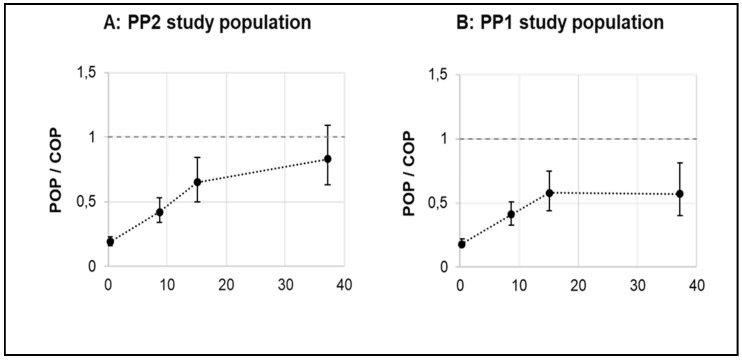
Ratio of total POP/total COP in serum with increasing dietary intakes of POP. Data are presented as mean plus 95% CI. Total COP and POP refer to the sum of all COP and POP measured including 7-keto and 7α/β-OH derivates of PS and cholesterol, respectively. Stabilized total POP concentration refers to the steady-state concentration as modelled over the six-week intervention. For PP2 study population (**A**): All study participants, *n* = 52 (*n* = 14 in control, *n* = 15 in low POP dose, *n* = 13 in medium POP dose and *n* = 10 in high POP dose group). For PP1 study population (**B**): All study participants, *n* = 59 (*n* = 14 in control, *n* = 15 in low POP dose, *n* = 15 in medium POP dose and *n* = 15 in high POP dose group).

The serum COP concentration served as a reference for the assessment of serum POP concentration after increased dietary intake of POP. Therefore, the ratio of POP/COP was calculated as presented in Figure 5**.** For the PP2 study population, the mean (plus 95% CI) POP/COP ratio was 0.19 (0.16–0.23), 0.42 (0.34–0.53), 0.65 (0.5–0.84), and 0.83 (0.63–1.09), respectively, in the control, low-dose, medium-dose, and high-dose POP groups. For the PP1 study population the mean (plus 95% CI) POP/COP ratio was 0.19 (0.16–0.23), 0.42 (0.34–0.53), 0.58 (0.44–0.75), and 0.57 (0.40–0.81), respectively, in the control, low-dose, medium-dose, and high-dose POP groups.

### 3.7. Composition of Serum Individual POP and COP after Dietary Intake of Increasing POP Doses

Baseline and stabilised serum concentrations of individual and total POP and COP as well as cholesterol standardized concentrations are shown in Table 3 for the PP2 study population and in Table S1 for the PP1 study population.

The PP2 study population shows no differences between groups in terms of the serum POP and COP concentrations as well as the composition of individual POP and COP at the baseline (Table 3). In the control group, serum POP and COP composition did not change during the intervention period. With increasing intakes of POP, there was a tendency towards a decrease in the relative abundance of 7-keto-PS, while the relative abundance of 7β-OH-PS increased (Table 3). Consumption of the three POP doses did not affect the COP composition compared to either the baseline or the control. For serum COP, 7α-OH-cholesterol was the predominant individual COP. In contrast, 7-keto-PS (sum of 7-keto sitosterol and 7-keto campesterol) were the most dominant individual POP. Notably, the composition of individual serum POP after POP intake was apparently different from the composition of individual POP in the study cookies, which were mainly composed of 7β-OH-PS and 7-keto-PS (Table 1).

In the PP1 study population, the composition of individual serum POP and COP in the control and the POP dose groups was similar to the findings in the PP2 study population (Table S1). Stabilised absolute and cholesterol-standardised concentrations of total and individual POP in the medium-dose and high-dose groups were lower than their corresponding concentrations in the PP2 study population.

### 3.8. Serum PS Concentrations after Daily Intake of PS and Increasing Dietary Intakes of POP 

As the PS intake in the three POP dose groups was similar (on average ca. 3 g/d), effects on serum PS concentrations compared to the control were assessed in the combined POP dose groups. At the baseline, serum sitosterol and campesterol and total PS concentrations were comparable between the control and combined POP dose groups sitosterol 7.07 ± 2.29 vs. 8.37 ± 4.46 μmol/L, campesterol 11.0 ± 3.7 vs. 14.3 ± 7.7 μmol/L and total PS 18.1 ± 5.8 vs. 22.6 ± 12.0 μmol/L, (all mean ± SD)). After six weeks, serum sitosterol concentration was significantly increased (*P* < 0.01) in the POP dose groups by on average 7.37 µmol/L (95% CI: 5.83; 8.91 µmol/L), while the serum campesterol concentration non-significantly increased by 0.93 µmol/L (95% CI: ‒0.89; 2.75 µmol/L) compared to the control. The absolute and relative increases of serum total PS were 7.88 µmol/L and 32.4%, respectively, compared to the control. Serum total PS accounted for <0.6% compared to the total sterol concentration. 

Similar findings for the increase in serum PS were observed for the PP1 study population, although absolute PS concentrations and the absolute and relative changes were slightly lower compared to those in the PP2 study population.

PS intake compared to the control resulted in a significant (*P* < 0.01) decrease in serum TC (5.85 mmol/L (all POP groups combined) vs. 6.28 mmol/L (control), as measured by the GC-FID method in the same run as for the PS analysis. This resulted in a relative decrease of serum TC of 6.1%.

### 3.9. Serum Concentrations of Cholesterol and TG after Daily Intake of PS and Increasing Dietary Intakes of POP 

Serum TC (–5%, *p* = 0.08) and LDL-C (−7%, *p* = 0.02), compared to the baseline were reduced in the control group. Compared to the control, the average ca. 3 g/d PS intake had no significant effects on serum TC and LDL-C, HDL-C, and TG concentrations. TC and LDL-C were non-significantly lowered by −2.5% (95% CI: −6.5; 1.6; *p* = 0.12) and -1.8% (95% CI: −6.7; 3.3; *p* = 0.30), respectively. Similar findings were found for the PP1 study population.

## 4. Discussion

This randomised placebo-controlled dose‒response study aimed to quantify the changes in serum POP concentrations after six weeks’ dietary intake of relatively high doses of POP (9–37 mg/d). For comparison, serum COP concentrations were also measured. Serum POP concentrations increased nonlinearly with intakes of increasing POP doses and reached stabilised concentrations after about one week. With all POP doses, the average POP/COP ratio remained <1.

Based on literature reviews [14,15] and data on POP contents of foods prepared through frying with PS-added margarines [18,19], the estimated daily intake of POP from such fried foods ranges from 0.3 to 64 mg/d, with a median intake of 7.4 mg/d POP [18,19]. Hence, the highest POP dose (37.2 mg/d) applied in this study falls within the estimated upper (90–95% quantile) daily POP intake (25–48 mg/d), which may represent an occasional POP exposure through the consumption of fried POPs prepared with a PS-added margarine. The lowest POP dose (8.7 mg/d) more closely resembles the estimated median daily POP intake and therefore seems to represent a more realistic sustained daily POP intake from foods fried with PS-added margarines.

While circulating POP concentrations have been measured after human consumption of PS-added margarines with PS and POP intakes of 3 g/d and <1 mg/d, respectively [16,17], this is the first study to investigate changes in serum POP concentrations after intakes of increasing doses of POP up to 37 mg. In the six-week intervention, serum POP concentrations increased nonlinearly and reached a plateau at a dose >15 mg/d. Based on steady-state modelling, stabilized POP concentrations were reached in one week and remained stable during the following weeks, indicating that a new steady state was reached rapidly. The underlying mechanism that may explain this rapid elimination of POP from the circulation, as shown for PS [33], is via increased biliary secretion into the gut [34]. Furthermore, Schött et al. [35] demonstrated both in humans and in a mouse model that POP were not preferentially transported from the blood into tissues. Interestingly, reported COP concentrations (up to 7.7 µmol/L) in human hepatic bile [36] are about 30 times higher than the serum COP concentrations (223−262 nmol/L) we report here after POP intake. These observations together suggest that biliary secretion of oxidized sterols is sufficient to control further rises in plasma/serum concentrations, although this has not yet been shown specifically for POP.

Serum POP concentrations are presented for both the PP1 and PP2 study population. Data from the PP2 study population represent the most conservative (“worst-case”) interpretation of the effect of daily intakes of increasing POP doses on serum POP concentrations and were defined as the main analysis outcome, so we primarily focus on these findings. At the baseline, serum POP concentrations were similar across all study groups, ranging between 36.8 and 40.8 nmol/L. These concentrations are higher than the reported POP concentrations of 12 nmol/L from two studies with healthy individuals consuming 0.51–0.68 mg/d of POP [16,17]. There is no clear explanation for this discrepancy, which might be caused by differences in the background diet of the study population. More recently, Fuhrmann et al. [37] and Baumgartner et al. [38] showed that the mean POP concentrations in two large cohorts (400‒500 subjects) were 22‒33 nmol/L, which is within the range of the data obtained in our study. These studies [37,38] further showed that none of the investigated POP, except for 7α-OH-campesterol and the 7α-OH-campesterol-to-cholesterol ratio in the Fuhrmann et al. [37] study, showed an association with cardiovascular events.

In the control group with a minimal POP intake of 0.3 mg/d, serum POP concentrations of about 39 nmol/L remained stable over time. Relative to the control, daily POP intakes up to 15 mg/d linearly increased serum POP concentrations, while the further increase in POP intake to 37 mg/d only had a small additional effect. The stabilised mean (± SD) POP concentrations after high-dose POP intake were 203 ± 64 and 150 ± 93 nmol/L in the PP2 and PP1 study populations, respectively. These concentrations are much lower than the reported serum/plasma POP concentrations of 17.3–79.6 μmol/L in apoE-/- mice after ingestion of 200 mg POP/kg diet for nine weeks [9], or via intraperitoneal injection with 1 mg/d POP (in the form of synthesized 7β-OH-sitosterol) for four weeks [39]. Even with such extreme POP concentrations, no acceleration in atherosclerosis was observed in these mice [9,39]. Hence, it seems unlikely that the observed increases in POP concentrations in our study may cause adverse effects. Furthermore, Schött et al. [35] demonstrated that there was no apparent correlation between POP concentrations in plasma and tissue, specifically aortic valve cusps, which would further mitigate any putative increase in CVD risk in humans. Lastly, even at the highest POP intake, POP concentrations remained about 3000 times lower than circulating cholesterol concentrations and about 20 times lower than serum PS concentrations, making it unlikely that POP may meaningfully contribute to atherosclerosis.

Mean serum COP concentrations (sum of 7-keto- and 7α/β-OH-CH) ranged in this study from 217 to 267 nmol/L, and remained stable over the intervention period, with no differences between the control and POP dose groups. Thus, COP concentrations were not affected by an increased POP (or PS) intake and increased serum POP concentrations. The serum COP concentrations measured in our study are comparable to those (sum of 7-keto-CH and 7α/β-OH-CH) reported by Zieden et al. [23] and those reviewed by Kulig et al. [24].

COP have been associated with atherogenicity and an increased CVD risk [6]. Similar to COP, POP are suggested to be atherogenic, but were shown to be less cytotoxic than COP at comparable molar concentrations [40,41,42]. Therefore, serum COP concentrations can be considered as a reference for the hazard assessment of serum POP. While with increasing POP doses the POP/COP ratio also increased compared to the control, the mean (and 95% CI) ratios in the low-dose and medium-dose POP groups remained lower than 1. Only in the high-dose POP group did the upper 95% CI exceed a ratio of 1 in the PP2 (but not in the PP1 study population). This indicates that only in a small number of individuals (3/10 in PP2 and 3/15 in PP1 study population) serum POP concentrations after high-dose POP intake exceeded serum COP concentrations.

For serum COP, 7α-OH-CH was the most abundant individual COP, contributing 67‒74% to the total COP, followed by 7-keto-CH and 7β-OH CH. These findings are in line with those observed by Zieden et al. [23] and reviewed by Kulig et al. [24]. For POP, 7-keto-PS (referring to the sum of 7-keto sitosterol and 7-keto campesterol) were the most abundant individual POP at the baseline. In the control group, the relative abundance of 7-keto-PS remained stable over time at 75–77% of POP, while 7β-OH-PS and 7α-OH-PS contributed ~21% and ≤3.1%, respectively. The increased POP intake from the cookies resulted in a decrease in the relative abundance of 7-keto-PS (up to 52%) and a subsequent increase of 7β-OH-PS (up to 45%), with 7α-OH-PS remaining unchanged. Nevertheless, 7-keto-PS were still the most abundant POP. Similar POP compositions were also reported by Baumgartner et al. [16] and Husche et al. [17] in individuals receiving a much lower POP dose (0.5–0.7 mg/d) through consumption of non-heated margarines with added PS. The noted difference in the individual composition between COP and POP can be explained by their different origin, i.e., from exogenous (dietary) or endogenous (enzymatic and nonenzymatic oxidation) sources [43]. COP formation through endogenous oxidation seems to be the major source for circulating COP, while intake of dietary POP seems to be the major source of serum POP concentrations.

Daily intake of ~3 g/d PS for six weeks increased serum PS concentrations modestly, comparable to previous findings, as summarised by Ras et al. [12]. No significant effects of PS intake on TC and LDL-C (or HDL-C and TG) concentrations compared to the control were found. Based on a recent meta-analysis, a sustained daily PS intake of 3 g/d typically lowers LDL-C by about 10–12% [2]. The absence of such a cholesterol-lowering effect is therefore unexpected but may be partly explained by the decrease in LDL-C concentrations seen in the control group following the baseline measurements, possibly because of a change in the background diet during intervention. The fact that the study was not really powered to investigate the effect of PS intake on serum lipids is another obvious explanation. It is worth noting that, looking at the relative changes in LDL-C concentrations for the three POP dose groups separately compared to the control, no statistically significant differences were found between the three POP doses. This supports the idea that the increased POP intake did not lead to a diminishing of the cholesterol-lowering effect of PS.

A major strength of our study is its randomized, double-blind, placebo-controlled design, and that we have tested three increasing POP doses to reflect the range of realistic estimated dietary POP intakes from foods that may be prepared by frying with a PS-added margarine. A limitation is that it was an explanatory, pilot-type study and we did not collect dietary intake data prior to and during the study.

## 5. Conclusions

Daily intake of increasing POP doses increased serum POP concentrations nonlinearly and reached a steady state, i.e., a stabilised concentration, in <7 days. Serum POP concentrations reached a plateau with daily intake of >15 mg and remained below serum COP concentrations following daily intake of POP doses for six weeks in a range of realistic estimated dietary POP intakes from foods prepared by frying with PS-added margarines. Increasing dietary POP intake had no effect on serum COP concentration.

## Figures and Tables

**Table 1:** Contents of plant sterols (PS) and plant sterol oxidation products (POP) plus relative distribution of individual POP in the study products (margarines and cookies)

	Regular margarine *^a^*	PS-added margarine *^a^*	Cookies *^a^*			
			Control	Low-dose POP	Medium-dose POP	High-dose POP
**Total PS *^b^*** **g/100 g product**	0.11	9.35	0.1 ± 0.0	2.4 ± 0.1	4.9 ± 0.3	9.7 ± 0.5
**Total POP** **mg/100 g product**	0.27	1.81	1.1 ± 0.4	31.6 ± 1.1	55.9 ± 2.9	133.6 ± 13.3
**Individual POP**	**% of total POP**					
**7α-OH-PS**	7.1	11.0	4.1	4.8	4.1	4.5
**7β-OH-PS**	7.1	13.7	11.2	20.1	21.4	22.9
**7-keto-PS**	82.1	22.5	31.6	16.3	14.8	13.5
**5α,6α-epoxy-PS**	n.d.	9.3	13.3	16.4	16.5	16.5
**5β,6β-epoxy-PS**	n.d.	10.4	31.6	38.7	39.1	39.3
**3β,5α,6β-triol-PS**	3.6	33.0	8.2	3.7	4.1	3.2
**Sum**	100	100	100	100	100	100

Data are presented as mean ± SD. n.d. = not detectable *^a^* Data for the two margarines are based on the analysis of one sample; for cookies, data are based on the analysis of nine samples (three samples per batch of cookies baked). *^b^* Sitosterol and campesterol contributed with 64% and 13%, respectively to total PS. Stigmasterol, brassicasterol, sitostanol, campestanol, and other sterols together composed 23% of total PS. 7α-OH-PS = 7α-hydroxy PS, 7β-OH-PS = 7ß-hydroxy PS.

**Table 2:** Anthropometric characteristics and blood lipids of study participants at the baseline.

Characteristics *n* = 60 (47% Males)	Mean ± SD (range)
Age (years)	56.3 ± 7.8 (42.0–72.0)
Body weight (kg)	79.0 ± 17.2 (47.4–118.5)
Height (cm)	174.3 ± 10.1 (149–198)
BMI (kg/m^2^)	25.8 ± 3.9 (18.4–34.7)
**Blood lipids (all *n* = 59)**	**Mean ± SD**
TC (mmol/L) 6.3 ± 0.7	6.3 ± 0.7
LDL-C (mmol/L)	4.1 ± 0.6
HDL-C (mmol/L)	1.5 ± 0.3
TG (mmol/L)	1.4 ± 0.7
**Blood lipids per study group**	
Placebo (*n* = 14)	
TC (mmol/L)	6.3 ± 0.8
LDL-C (mmol/L)	3.6 ± 0.6
HDL-C (mmol/L)	1.7 ± 0.3
TG (mmol/L)	1.3 ± 0.5
Low-dose POP (*n* = 15)	
TC (mmol/L)	6.2 ± 1.0
LDL-C (mmol/L)	3.6 ± 0.7
HDL-C (mmol/L)	1.6 ± 0.4
TG (mmol/L)	1.6 ± 0.9
Medium-dose POP (*n* = 15)	
TC (mmol/L)	6.3 ± 0.8
LDL-C (mmol/L)	3.7 ± 0.5
HDL-C (mmol/L)	1.5 ± 0.3
TG (mmol/L)	1.6 ± 0.7
High-dose POP (*n* = 15)	
TC (mmol/L)	6.1 ± 0.7
LDL-C (mmol/L)	3.5 ± 0.4
HDL-C (mmol/L)	1.6 ± 0.3
TG (mmol/L)	1.4 ± 0.8

BMI: body mass index; TC: total cholesterol; LDL: low density lipoprotein, LDL-C: low-density lipoprotein cholesterol, HDL: high density lipoprotein, HDL-C: high density lipoprotein cholesterol TG: triglycerides, POP: plant sterols oxidation products.

**Table 3:** Absolute baseline and stabilized concentrations *^a^* and total cholesterol-standardized concentrations of total and individual POP (plant sterol oxidation products) and COP (cholesterol oxidation products) during a six-week dietary intake of increasing doses of POP (PP2 study population).

POP					COP				
	Control (*n* = 14)	Low-dose POP (*n* = 15)	Medium-dose POP (*n* = 13)	High-dose POP (*n* = 10)		Control (*n* = 14)	Low-dose POP (*n* = 15)	Medium-dose POP (*n* = 13)	High-dose POP (*n* = 10)
nmol/L ^*a*^ (with % of total POP in brackets)					nmol/L ^*a*^ (with % of total COP in brackets)				
**Baseline**									
**7α-OH-PS**	1.05 ± 0.37 ^*b*^ (2.7)	1.24 ± 0.60 (3.0)	1.23 ± 0.52 (3.2)	1.27 ± 0.40 (3.5)	7α-OH-CH	148.6 ± 71.5 (68.4)	151.8 ± 95.1 (67.2)	153.2 ± 67.8 (68.2)	197.4 ± 168.9 (73.5)
**7β-OH-PS**	8.05 ± 2.65 (20.5)	8.19 ± 2.95 (20.1)	8.20 ± 3.05 (21.3)	7.69 ± 1.43 (20.9)	7β-OH-CH	26.3 ± 5.2 (12.1)	28.5 ± 8.6 (12.6)	27.6 ± 3.9 (12.3)	28.1 ± 4.4 (10.5)
**7-keto-PS**	30.2 ± 5.6 (76.8)	31.3 ± 5.6 (76.7)	29.0 ± 5.7 (75.3)	27.7 ± 1.8 (75.3)	7-keto-CH	41.9 ± 5.0 (19.3)	45.4 ± 17.7 (20.1)	43.3 ± 6.7 (19.3)	42.8 ± 8.0 (15.9)
**Total POP**	39.3 ± 8.2	40.8 ± 8.8	38.5 ± 9.0	36.8 ± 2.6	Total COP	217.1 ± 73.9	226.0 ± 97.5	224.5 ± 67.2	268.7 ± 177.4
**Stabilized**									
**7α-OH-PS**	1.22 ± 0.40 (3.1)	2.43 ± 0.81 (2.7)	4.62 ± 1.77 (3.2)	6.72 ± 1.71 (3.3)	7α-OH-CH	156.2 ± 78.9 (70.2)	142.5 ± 81.2 (66.9)	164.4 ± 104.1 (71.4)	191.7 ± 133.2 (73.1)
**7β-OH-PS**	8.22 ± 2.51 (21.1)	32.0 ± 12.8 (35.2)	59.4 ± 18.4 (41.1)	91.4 ± 30.1 (45.0)	7β-OH-CH	24.8 ± 2.9 (11.1)	25.6 ± 7.9 (12.0)	24.3 ± 2.4 (10.6)	25.1 ± 2.5 (9.6)
**7-keto-PS**	29.3 ± 4.3 (75.3)	56.4 ± 15.8 (62.0)	80.1 ± 19.0 (55.5)	104.5 ± 34.0 (51.5)	7-keto-CH	40.70 ± 5.6 (18.3)	43.2 ± 13.7 (20.3)	40.4 ± 4.9 (17.5)	43.3 ± 6.8 (16.5)
**Total POP**	38.9 ± 6.9	91.0 ± 27.9	144.4 ± 37.9	203.0 ± 63.7	Total COP	222.5 ± 80.5	212.9 ± 84.6	230.2 ± 106.8	262.1 ± 138.4
**Change from baseline**									
**7α-OH-PS**	0.18 ± 0.32	1.19 ± 0.78	3.39 ± 1.77	5.45 ± 1.84	7α-OH-CH	7.62 ± 24.34	−9.26 ± 44.52	11.2 ± 65.6	−5.67 ± 80.05
**7β-OH-PS**	0.18 ± 2.68	23.8 ± 11.7	51.2 ± 17.0	83.7 ± 29.4	7β-OH-CH	−1.43 ± 4.5	−2.83 ± 8.66	−3.31 ± 3.66	−3.03 ± 3.77
**7-keto-PS**	−0.87 ± 5.06	25.0 ± 13.9	51.1 ± 16.8	76.8 ± 33.6	7-keto-CH	−1.26 ± 4.95	−2.14 ± 18.09	−2.84 ± 5.41	0.48 ± 6.23
**Total POP**	−0.45 ± 7.76	50.2 ± 24.6	105.9 ± 34.1	166.2 ± 62.6	Total COP	5.39 ± 28.66	−13.1 ± 55.6	5.74 ± 71.54	−6.56 ± 85.43
**nmol/mmol total cholesterol**									
**Baseline**									
**7α-OH-PS**	0.17 ± 0.06	0.20 ± 0.10	0.20 ± 0.09	0.21 ± 0.09	7α-OH-CH	24.2 ± 11.9	25.0 ± 15.3	24.4 ± 11.5	32.0 ± 28.1
**7β-OH-PS**	1.29 ± 0.33	1.32 ± 0.51	1.31 ± 0.54	1.24 ± 0.23	7β-OH-CH	4.26 ± 0.72	4.56 ± 1.14	4.37 ± 0.57	4.57 ± 0.99
**7-keto-PS**	4.89 ± 0.65	5.13 ± 1.14	4.62 ± 1.11	4.49 ± 0.58	7-keto-CH	6.87 ± 1.07	7.30 ± 2.53	6.88 ± 1.24	6.98 ± 1.79
**Total POP**	6.35 ± 0.92	6.66 ± 1.63	6.14 ± 1.69	5.97 ± 0.77	Total COP	35.4 ± 12.3	36.9 ± 15.6	35.7 ± 11.8	43.6 ± 30.0
**Stabilized**									
**7α-OH-PS**	0.20 ± 0.06	0.43 ± 0.17	0.76 ± 0.29	1.20 ± 0.29	7α-OH-CH	25.0 ± 12.1	25.2 ± 14.5	26.4 ± 15.6	34.3 ± 23.9
**7β-OH-PS**	1.32 ± 0.32	5.65 ± 2.34	9.80 ± 3.12	16.3 ± 5.2	7β-OH-CH	4.02 ± 0.32	4.45 ± 1.27	4.02 ± 0.46	4.50 ± 0.55
**7-keto-PS**	4.76 ± 0.71	10.1 ± 3.5	13.2 ± 3.3	18.6 ± 5.4	7-keto-CH	6.63 ± 1.07	7.55 ± 2.39	6.69 ± 0.97	7.78 ± 1.43
**Total POP**	6.29 ± 0.98	16.2 ± 5.7	23.8 ± 6.5	36.1 ± 10.5	Total COP	35.8 ± 12.1	37.5 ± 15.3	37.3 ± 15.6	47.0 ± 24.9
**Change from baseline**									
**7α-OH-PS**	0.03 ± 0.05	0.23 ± 0.15	0.56 ± 0.28	0.99 ± 0.32	7α-OH-CH	0.82 ± 4.5	0.21 ± 7.78	2.02 ± 10.73	2.35 ± 13.65
**7β-OH-PS**	0.03 ± 0.39	4.33 ± 2.19	8.5 ± 2.94	15.1 ± 5.1	7β-OH-CH	−0.25 ± 0.6	−0.11 ± 1.51	−0.35 ± 0.52	−0.07 ± 0.91
**7-keto-PS**	−0.13 ± 0.68	4.92 ± 3.15	8.6 ± 2.94	14.1 ± 5.4	7-keto-CH	−0.24 ± 0.76	0.25 ± 3.01	−0.19 ± 0.99	0.79 ± 1.38
**Total POP**	−0.06 ± 1.08	9.51 ± 5.17	17.7 ± 5.9	30.1 ± 10.3	Total COP	0.43 ± 5.06	0.54 ± 9.98	1.59 ± 11.72	3.38 ± 14.87

*^a^* Stabilized concentrations were derived from all available data from days 14 to 42. A similar procedure was performed for baseline concentrations that are based on days ‒2 and 0. Change from baseline refers to stabilized minus baseline concentration. *^b^* Data are presented as mean ± SD.3.7. Ratio of POP/COP with Increasing Dietary POP Intakes

## Supplementary Materials

The following are available online at https://www.mdpi.com/2072-6643/11/10/2319/s1 Table S1. Absolute baseline and stabilized concentrations and total cholesterol-standardized concentrations of total and individual POP and COP during 6-week dietary intake of increasing doses of POP (PP1 study population).
