# Supplementary material for: Serum Concentration of Plant Sterol Oxidation Products (POP) Compared to Cholesterol Oxidation Products (COP) after Intake of Oxidized Plant Sterols: A Randomised, Placebo-Controlled, Double-Blind Dose–Response Pilot Study
Source: Nutrients. 2019 Sep 30;11(10):2319. doi: 10.3390/nu11102319 (PMC6835335; doi:10.3390/nu11102319)
Supplement: Supplementary file 1 [file nutrients-11-02319-s001.pdf]

Supplementary Materials

Table S1. Absolute baseline and stabilized concentrations[a] and total cholesterol-standardized concentrations of total and individual POP and COP during 6-week dietary intake of increasing doses of POP (PP1 study population).

	**POP**					**COP**			
	**Control (*n* = 14)**	**Low-dose POP (*n* = 15)**	**Medium-dose POP (*n* = 15)**	**High-dose POP (*n* = 15)**		**Control (*n* = 14)**	**Low-dose POP (*n* =15)**	**Medium-dose POP (*n* = 15)**	**High-dose POP (*n* = 15)**
	**nmol/L[a] (% of total POP)**					**nmol/L[a] (% of total COP)**			
**Baseline**									
7α-OH-PS	1.05±0.37[b] (2.7)	1.24±0.6 (3.0)	1.15±0.53 (3.0)	1.29±0.57 (3.0)	7α-OH-CH	148.6±71.5 (68.4)	151.8±95.1 (67.1)	150.8±63.1 (67.7)	176.0±143.4 (71.5)
7β-OH-PS	8.05±2.65 (20.5)	8.19±2.95 (20.1)	7.99±3.01 (21.1)	9.84±6.99 (22.6)	7β-OH-CH	26.3±5.2 (12.1)	28.5±8.6 (12.6)	27.9±4.4 (12.5)	27.6±3.8 (11.2)
7-keto-PS	30.2±5.6 (76.8)	31.3±5.6 (76.7)	28.6±5.5 (75.7)	32.1±12.3 (73.8)	7-keto-CH	41.9±5.00 (19.3)	45.4±17.7 (20.1)	43.6±6.8 (19.6)	42.3±6.9 (17.2)
Total POP	39.3±8.3	40.8±8.8	37.8±8.8	43.6±19.9	Total COP	217.1±73.9	226.0±97.5	222.8±62.7	246.3±151.0
**Stabilized**									
7α-OH-PS	1.22±0.4 (3.1)	2.43±0.81 (2.7)	4.21±2.01 (3.2)	4.92±2.99 (3.3)	7α-OH-CH	156.2±78.9 (70.2)	142.5±81.2 (66.9)	157.1±98.6 (70.6)	178.9±124.3 (72.3)
7β-OH-PS	8.22±2.51 (21.1)	32.0±12.8 (35.2)	52.9±24.3 (40.4)	64.1±46.7 (42.7)	7β-OH-CH	24.8±2.9 (11.2)	25.6±7.9 (12.0)	24.1±2.6 (10.8)	24.7±2.4 (10.0)
7-keto-PS	29.3±4.3 (75.4)	56.4±15.8 (61.9)	73.6±24.8 (56.2)	80.8±44.5 (53.8)	7-keto-CH	40.7±5.6 (18.3)	43.2±13.7 (21.3)	40.3±4.6 (18.1)	42.4±6.3 (17.1)
Total POP	38.9±6.9	91.0±27.9	130.9±50.1	150.2±93.1	Total COP	222.5±80.5	212.9±84.6	222.4±101.2	247.6±130.0
**Change from baseline**									
7α-OH-PS	0.18±0.32	1.19±0.78	3.05±1.89	3.62±3.07	7α-OH-CH	7.62±24.34	−9.26±44.52	6.28±62.85	2.9±70.6

7β-OH-PS	0.18±2.68	23.8±11.7	44.9±23.0	54.3±49.3	7β-OH-CH	−1.43±4.5	−2.83±8.66	−3.83±3.91	−2.97±3.25
7-keto-PS	-0.87±5.06	25.0±13.9	44.9±22.6	48.7±49.7	7-keto-CH	−1.26±4.95	−2.14±18.09	−3.33±5.57	0.09±5.14
Total POP	-0.45±7.76	50.2±24.6	93.1±46.3	106.6±101.5	Total COP	5.39±28.66	−13.1±55.6	−0.41±69.5	1.28±74.6
nmol/mmol cholesterol									
**Baseline**									
7α-OH-PS	0.17±0.06	0.2±0.1	0.18±0.09	0.21±0.11	7α-OH-CH	24.2±11.9	25.0±15.3	23.9±10.8	28.6±23.7
7β-OH-PS	1.29±0.33	1.32±0.51	1.26±0.52	1.59±1.17	7β-OH-CH	4.26±0.72	4.56±1.14	4.37±0.55	4.52±0.85
7-keto-PS	4.89±0.65	5.13±1.14	4.53±1.05	5.23±2.02	7-keto-CH	6.87±1.07	7.3±2.53	6.88±1.15	6.93±1.56
Total POP	6.35±0.92	6.66±1.63	5.99±1.61	7.08±3.32	Total COP	35.4±12.3	36.9±15.6	35.3±11.0	40.2±25.3
**Stabilized**									
7α-OH-PS	0.2±0.06	0.43±0.17	0.69±0.33	0.88±0.53	7α-OH-CH	25.0±12.1	25.2±14.5	25.5±14.8	32.0±21.9
7β-OH-PS	1.32±0.33	5.65±2.34	8.73±4.06	11.4±8.2	7β-OH-CH	4.02±0.32	4.45±1.27	3.99±0.44	4.45±0.48
7-keto-PS	4.76±0.71	10.1±3.5	12.2±4.1	14.4±7.5	7-keto-CH	6.63±1.07	7.55±2.39	6.71±0.92	7.64±1.22
Total POP	6.29±0.98	16.2±5.7	21.6±8.4	26.8±16.1	Total COP	35.8±12.1	37.5±15.3	36.4±14.8	44.4±22.9
**Change from baseline**									
7α-OH-PS	0.03±0.06	0.23±0.15	0.51±0.3	0.67±0.54	7α-OH-CH	0.82±4.5	0.21±7.78	1.61±10.08	3.4±12.06
7β-OH-PS	0.03±0.4	4.33±2.19	7.47±3.86	9.85±8.67	7β-OH-CH	−0.25±0.6	−0.11±1.51	−0.37±0.5	−0.07±0.76

7-keto-PS	0.13±0.68	4.92±3.15	7.63±3.73	9.16±8.43	7-keto-CH	−0.24±0.76	0.25±3.01	−0.17±0.95	0.7±1.18
Total POP	0.06±1.08	9.51±5.17	15.6±7.7	19.7±17.5	Total COP	0.43±5.06	0.54±9.98	1.15±11.06	4.26±12.93

[a]Stabilized concentrations were derived from all available data from days 14 to 42. A similar procedure was performed for baseline concentrations [b]Data are presented as mean ± SD.
